# Two-Dimensional Preoperative Digital Templating is Less Accurate When Using a Collared Triple Taper Stem Versus a Single Taper Design

**DOI:** 10.1016/j.artd.2025.101658

**Published:** 2025-03-11

**Authors:** Claudio Diaz-Ledezma, Angel X. Xiao, Juan David Lizcano, Erik N. Hansen, Camilo Restrepo, William J. Hozack

**Affiliations:** a Department of Orthopedic Surgery, University of California San Francisco, San Francisco, CA, USA; b Rothman Orthopaedic Institute at Thomas Jefferson University, Philadelphia, PA, USA

**Keywords:** Templating, Triple taper stems, Total hip arthroplasty

## Abstract

**Background:**

Collared triple taper stems (CTTS) and single taper stems (STS) have an excellent performance in primary total hip arthroplasty (THA). While 2-dimensional radiographic templating is accurate for STS, data are lacking for CTTS. We hypothesize that CTTS’ more anatomical design in the anteroposterior dimension may lead to inaccurate templating sizing. This study compared templating accuracy of CTTS to a predecessor STS in THA patients.

**Methods:**

106 THA performed with CTTS were compared to 106 THA performed with STS by 2 high-volume surgeons. The stems chosen for comparison were manufactured by the same company, use the same templating software, shared an identical medial-lateral profile, and offered the same size range. The ability of digital templating to predict final implant size was evaluated.

**Results:**

Template to stem accuracy was 36.8% for CTTS and 49.1% for STS (*P* = .07). Accuracy within 1 size was 88.7% for CTTS versus 95.2% for STS (*P* = .1). CTTS was implanted using a smaller size compared to the template twice as frequently as STS (43.4% vs 20.8%; *P* < .01). CTTS was 3.7 times more likely to have implants 2 or more sizes under the template compared to STS (10.4% vs 2.8%; *P* = .02). In logistic regression, the only predictor of implant 2+ sizes under the template was type of stem (*P* = .04).

**Conclusions:**

The accuracy of conventional templating for CTTS is lower than the predecessor STS, with the template often suggesting a larger size. Bi-planar or 3-dimensional preoperative templating could potentially be a more accurate technique, especially during the initial learning curve with these stems.

## Introduction

For successful clinical outcomes with the introduction of a new stem design for total hip arthroplasty (THA), surgeons must recognize the importance of predictable preoperative planning combined with consistent and reproducible surgical execution. Recent data have proved that collared stems have an excellent performance with a lower rate of early failures compared to other designs [[1], [2], [3], [4], [5]]. Consequently, collared triple taper stems (CTTS) have been introduced in the market and are supported by promising registry [6] and institutional data [7] with satisfactory individual experiences using direct anterior approach (DAA) [5,8], mini posterior [9], and posterior approach [5].

Girgis [10] indicated that “digital templating with 2-dimensional (2D) radiographs is likely the most cost-effective and efficient” form of preoperative planning. Uncemented stems have been defined as having accurate templating when the implant is “within 1 size” compared to the preoperative radiographic planning [11,12]. The 2 potential ramifications of a mismatch between template size and actual implant size chosen range from the concern that implanting a “template-undersized” stem may lead to fibrous ingrowth and loosening, while using the “template-sized” implant may be too large for the patient and lead to periprosthetic fracture.

CTTS designs offer a more anatomic filling in the anteroposterior (AP) plane of the proximal femoral metaphysis compared to single taper stems (STS), which mainly relies on medial-lateral filling of the stem to achieve intraoperative axial and rotational stability [13]. 2D radiographic digital templating, probably the most frequent type of preoperative planning nowadays, does not address the AP filling of the proximal femoral metaphysis. Furthermore, there are no published data, to the best of our knowledge, on the accuracy of 2D preoperative digital templating for CTTS.

The purpose of this study is to compare the accuracy of 2D radiographic digital template between a CTTS design and its predecessor STS design among patients who successfully underwent THA through DAA. The null hypothesis is that 2D radiographic digital templating is equally accurate for both stems.

## Material and methods

This is a retrospective, bi-institutional study conducted across 2 different academic centers, and approved by the Institutional Review Board (IRB #20-31690), employing clinical records and imaging data.

According to a recent study by Crutcher et al. [14], which used the same STS employed as a control in our study (Accolade II, Stryker, Mahwah, New Jersey), the accuracy rate of exact size between the 2D digital template and the actual implanted stem was 52.9%. Using that information, we decided that a difference of 20% less accuracy to the exact size would be clinically relevant. Using an alpha of 0.05 and beta of 0.2, the sample size required to demonstrate a difference is 105 cases per group. Sample size calculation resource can be found at https://sample-size.net/sample-size-proportions/.

A total of 212 hips from 201 patients (11 bilateral cases) who underwent uncomplicated THA through a previously described DAA technique [15] by 2 high-volume academic surgeons were included in the study. Institution A employed intraoperative fluoroscopic guidance during all surgeries, while Institution B did not use intraoperative fluoroscopy or radiographs. Cases were sequentially selected from each institution’s database. An uncomplicated THA was defined as a patient who did not present any intraoperative complications, plus absence of any reintervention or reoperations, including closed reduction for dislocation, during the first 6 weeks.

A total of 106 THA procedures performed with CTTS (Insignia, Stryker, Mahwah, New Jersey) were compared to 106 THA procedures performed with STS (Accolade II, Stryker, Mahwah, New Jersey) between 2018 and 2023. These 2 stems were chosen for comparison because (1) they are produced by the same manufacturer, (2) the templated sizes were generated using the same image software, (3) the medial-lateral profile of the 2 stems is the same, and (4) they have the same number of sizes. The first 20 patients operated by each surgeon using CTTS were not included as we considered them part of the learning curve if we extrapolate data for implants developed by the same manufacturer using the same surgical approach [16]. The CTTS used in this study has a broach with 3 different tooth geometries that compacts bone in the AP plane, extracts bone mediolaterally, and a cutting profile at its most distal part [17]. The STS used in this study, on the contrary, uses a traditional extraction broach. In all cases, only the manufacturer-provided broaches were used to prepare the proximal femur by manual technique. Distal reamers were not used in any case to increase the size of the diaphyseal femoral canal.

Several exclusion criteria were applied. The number of patients excluded for each reason is described in parentheses. In total, 44 cases were excluded from the analysis.

(a) History of metabolic bone disease or conditions that may secondarily affect bone quality (eg, renal transplant, chronic renal disease, hyperparathyroidism) for whom the use of uncemented stems is arguable (1 case),
(b) patients with Crowe type II or higher degrees of hip dysplasia [18], or coxa valga exceeding 140° [19] (in whom the use of a tapered stem might be debatable) (1 case),
(c) patients undergoing conversion from a previously failed hip arthroplasty,
(d) patients for whom the preoperative template was created using radiographs from outside institutions. This exclusion minimized variability in pelvic and lower limb rotation, as well as magnification inconsistencies (3 cases),
(e) patients who experienced intraoperative femoral fractures requiring cerclage wires (1 case),
(f) patients who demonstrated radiographic subsidence exceeding 1.5 mm, a magnitude reported as relevant for short-term survivorship [20] within the first 6 weeks postoperatively (1 case),
(g) patients who underwent femoral stem revision due to aseptic loosening or periprosthetic fracture at the most recent clinical evaluation (1 case diagnosed with aseptic loosening but not yet revised),
(h) patients deceased at the time of the last chart review (3 cases), and
(i) cases in which the acetabular template was off by more than ± 1 size (pre-established as negative controls) (21 cases). One of the participating institutions contributed an additional 11 cases that were excluded to achieve balanced groups based on stem type by institution.

Demographic data collected included age, gender, height, weight, body mass index (BMI) (as a continuous and categorical value, categorizing as obese those patients with BMI ≥ 30 kg/m^2^), and the operated side (Table 1).

**Table 1:** Demographics.

Variable	Collared triple taper stem	Single taper stem	*P* value
Age in years (range)	62.8 (41-89)	67.2 (40-89)	.03
BMI in kg/m^2^ (range)	29.1 (21-47)	27.7 (18-39)	<.01

Variable	N	%	N	%	
Obese (BMI > 30 kg/m^2^)	28	26.4	28	26.4	1
Females	64	60.4	68	64.2	.6
Dorr A	17	16.0	26	24.5	.1
Dorr B	83	78.3	72	67.9	.08
Dorr C	6	5.7	8	7.5	.5
Side (left)	52	49.0	48	45.2	.5

BMI, body mass index; N, number; %, percentage.

### Radiographic evaluation and templates

The digital preoperative templates created by different adult reconstruction fellows and their attending were retrieved from the image repository at each institution. The software used were Traumacad (BrainLab Inc., Westchester, IL) at institution A and Sectra (Linkoping, Sweden) at institution B. All templates were performed in an AP pelvis view using a radiographic marker, following the recommendations described by González Della Valle [21] and Bayne [22]. During the study period, Institution A and Institution B did not change their radiographic technique, markers, or software. In addition, during the present study, 3 independent orthopaedic surgeons re-evaluated the templates to check for proper size and positioning. All of them were formally trained in THA templating using a video tool that has already been proved to enhance template quality [23]. The preoperative radiograph was used to classify the proximal femur anatomy according to Dorr’s classification (Table 1). The immediate postoperative radiograph was assessed for the evaluation of the collar position relative to the calcar. The 6-week radiograph was assessed for the detection of subsidence.

The implant data were retrieved at the operative report and confirmed within the electronic medical records to avoid misinformation [24]. It included type of stem, size, offset, bearing couple, head size and length, and acetabular components size.

Descriptive and statistical analysis was conducted using SPSS. The accuracy of templating compared to the final implant size was analyzed using Chi-square tests according to Crutcher’s method [14]. The differences between template size and stem size were categorized as follows: 2 or more sizes bigger, 1 size bigger, same size, 1 size smaller, and 2 or more sizes smaller.

## Results

**Figure 1:**
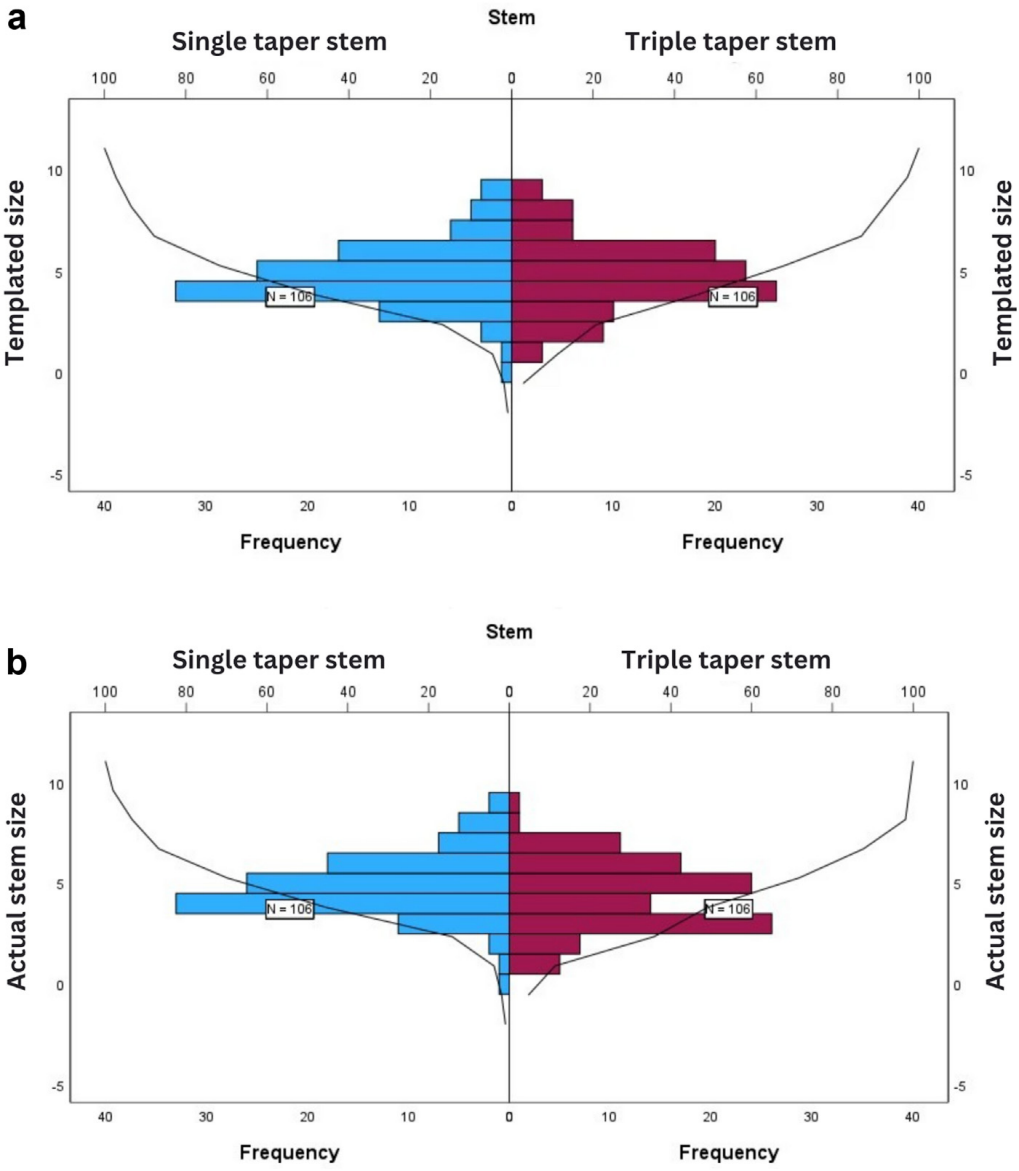
(a) Distribution of the templated sizes for both stems. (b) Distribution for the actual implanted sizes for both stems.

The CTTS had a median size [range] of 5 [[1], [2], [3], [4], [5], [6], [7], [8], [9]], and the STS had a median size [range] of 5 (0-9), with no significant difference between groups after using an independent-sample median test (*P* = .8). The final stem sizes for the whole sample were not normally distributed, thus Kolmogorov-Smirnov test was used for comparison between both stems. Although the distribution of the templated sizes was the same across categories of stems (*P* = .9), the actual implanted stem sizes did not have the same distribution between groups (*P* = .01) (Fig. 1). The CTTS have significantly more cases concentrated in “the smaller than the template” area.

The accuracy between the templated size and the implanted stem and the statistical value for each comparison are shown in Table 2. Exact matching of template to stem size was 36.8% for CTTS and 49.1% for STS (*P* = .07). Matching of template to stem size “within 1 size” was 88.7% for CTTS versus 95.2% for STS (*P* = .1). CTTS were implanted using a smaller size compared to the template twice as frequently as STS (43.4% vs 20.8%; *P* < .01). The CTTS group was 3.7 times more likely to have implants 2 or more sizes under the template compared to the STS group (10.4% vs 2.8%; *P* = .02).

**Table 2:** Accuracy between template and actual stem size.

Template stem size difference	Collared triple taper stem		Single taper stem		*P* value
	n	%	n	%	
2 or more sizes bigger	1	0.9	2	1.9	.5
1 size bigger	20	18.9	31	29.2	.07
Same size	39	36.8	52	49.1	.07
1 size smaller	35	33.0	18	17.0	**<.01**
2 or more sizes smaller	11	10.4	3	2.8	**.02**
Total	106	100.0	106	100.0	

Stem feature	n	%	n	%	*P* value
Bigger than the template	21	19.8	33	31.1	.08
Smaller than the template	46	43.4	21	19.8	**<.01**
Within 1 size	94	88.7	101	95.3	.1

n, number; %, percentage.

Bold values indicate statistical significance (*P* < .05).

High offset was employed in 72 CTTS cases (67.9%), and in 75 STS cases (70.8%), with no significant difference. Seventy-one cases (66%) of the CTTS group have the collar resting on the calcar at the immediate postoperative radiographs. The median cup size was 52 mm for both groups, with the same range of 46 to 60 mm. There was no statistical difference.

The use of fluoroscopic guidance did not demonstrate any significant difference in terms of accuracy for all included cases (*P* = .73) or for the possibility of the implant being bigger, equal to, or smaller than the template (*P* = .62). Analyzing the stems separately, neither the CTTS nor the STS showed significant differences when comparing the accuracy of the template being within 1 size with or without fluoroscopic guidance (*P* = .16 and *P* = .22, respectively).

Using binary logistic regression, controlled for age, obesity, Dorr’s classification, gender, and type of stem, the only predictor of “stems that were 2 or more sizes under the template” was the type of stem—CTTS (*P* = .04). Specifically examining CTTS, no association was observed between *“having the collar resting on the calcar”* and the size of the template being *smaller/equal/bigger* than the actual stem (*P* = .9), or the probability of having a template with 2 or more sizes under the actual stem (*P* = .4).

**Figure 2:**
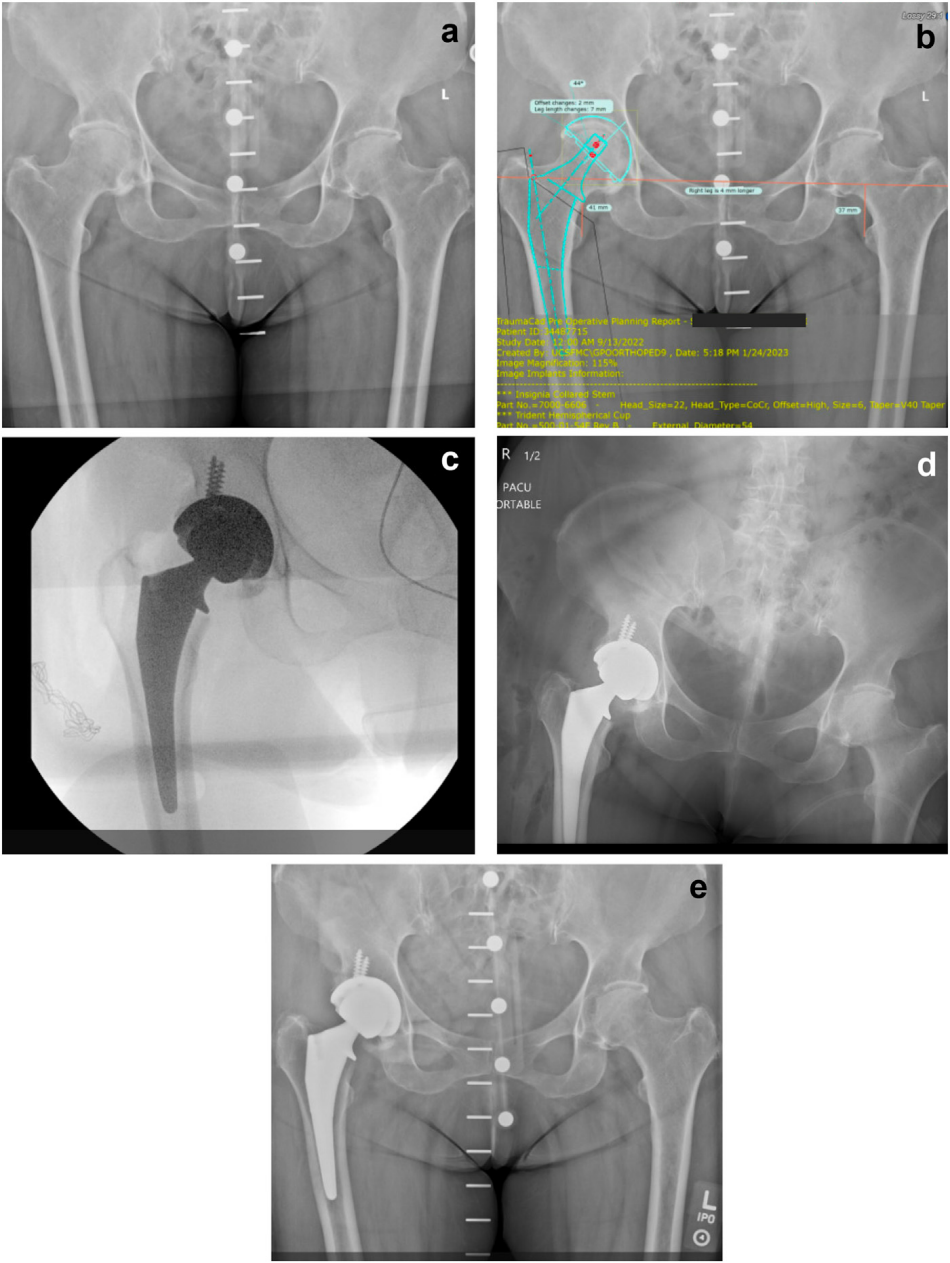
Case with 2 sizes under the template with a collared triple taper stem in a 75-year-old female. (a) Preoperative radiograph; (b) Preoperative template; (c) Intraoperative fluoroscopy; (d) Immediate postoperative radiograph; (e) 6 weeks postoperative radiograph.

Figure 2 shows an example with a template/stem difference of 2 sizes for CTTS.

## Discussion

Our study found differences in radiographic 2D preoperative planning for CTTS compared to STS. While both groups had a relatively high percentage of templates predicting stem size within 1 size of stem implanted, matching of the template to the exact stem size was observed in one-third of the CTTS and half of the STS. CTTS stems were twice as likely to be smaller than the templates than STS stems and had 3.7 times more stems that were 2 sizes under the template.

Three possible explanations exist for the observed difference in template accuracy between the 2 implants: (1) broach teeth design, (2) the presence of a collar, and (3) stem design. Published literature reports no difference in the clinical and radiographic results comparing extraction versus compaction broaches for uncemented stems [25,26]. The recently introduced hybrid broaches used for CTTS (compacting in the AP plane and extraction in the mediolateral plane) have not been studied in depth in clinical scenarios. Carlson et al. [27] demonstrated that the effort exerted by the surgeon to broach the femur and the seating height of the definitive implant are not different between the 2 stems used in our study. Studies assessing the accuracy of preoperative templating with stems using compaction broaches reported an accuracy within 1 size of up to 89% [28,29]. These findings are similar to our results with the CTTS. A study evaluating the accuracy within 1 size of template with the same STS (extraction broach) used in our study was 94% [14] which is also similar to our findings. Therefore, while the difference in broach teeth design (hybrid compaction/extraction vs extraction only) between the 2 implants could contribute to the observed variations in template accuracy, we feel the evidence suggests that any effect would be slight.

A collar offers additional axial and torsional stability [30]. This feature must be taken into consideration when choosing the appropriate size for a given patient. There have been concerns regarding the potential of undersizing of the femoral stem due to the added rotational stability and resistance to subsidence provided by the collar [31]. While there are few studies evaluating the accuracy of radiographic templating of collared stems, Ashkenazi et al. compared postoperative radiographic and clinical outcomes between patients who underwent THA with either a collared or a noncollared Corail stem. They found that both stems had similar postoperative canal fill ratios as well as comparable radiographic outcomes in terms of implant stability, fixation, and bone ingrowth [32]. In our study, both surgeons focused their surgical technique on obtaining both axial and rotational press fit for the broach as a priority. Once achieved, only then was calcar reaming performed for the CTTS stem to allow the collar to seat at the time of stem insertion. As such, this method aimed to minimize differences in the accuracy of the digital template adjusted by the presence or absence of a collar resting on the calcar.

The design of CTTS, which enhances metaphyseal filling of the proximal femur in the AP dimension, is the likely reason that a radiographic template based solely on the coronal plane is less accurate in predicting the implant size chosen by the surgeon during the operation, as compared to the STS design. The stems for this comparison study are produced by the same manufacturer, using the same image database, and have the same number of sizes, and most importantly, have the same medial-lateral profile. The main difference is in the AP metaphyseal dimension with the CTTS being more robust. A study by Issa et al.^24^ [33] described the medial-to-lateral radiographic fit and fill of the STS employed in our study. A 96% radiographic canal filling rate at 10 mm proximal to the lesser trochanter and a 90% rate at 6 cm distal to it were found. More recently, using the same methodology by Rainey et al. [8] found lower canal filling rates for the CTTS prosthesis: 85% at 10 mm proximal and 75% at 6 cm distal to the lesser trochanter. According to the current standards, the 2D template aims for canal filling as close as 100% in the proximal metaphysis. We found that 95% of templates were within 1 size fit for STS compared to 88% for the CTTS. An accurate template (within 1 size of the final implant) is a proxy of the concept of the canal filling rate pursued by the preoperative template, so the differences observed by Issa et al. and Rainey et al. may support our findings. In our view, the axial plane needs to be evaluated when attempting to predict the size needed during surgery, which is a primary objective of preoperative templating. This observation underscores the potential value of 3-dimensional (3D) templating for these types of stems (such as using computed tomography scans). The 3D planning concept has been probed to be an excellent alternative with other stem designs [34,35].

We believe including 2 surgeons using the same surgical approach with and without fluoroscopy strengthens the generalizability of our findings. Fluoroscopic guidance did not influence the observed results in our study. This matches a previous study from our institution [36]. Intraoperative fluoroscopy is often used for cup preparation, cup insertion, and calculation of offset and leg length changes. When used on the femoral side, it is generally to confirm fit only in the mediolateral dimension only.

Our study has limitations and strengths. First, we encountered a difference in age and BMI between the 2 groups. However, from a clinical standpoint, it is difficult to evaluate the influence of these differences in a study comparing the accuracy of a radiographic template. Additionally, when a multivariate analysis was run, these 2 mentioned variables lost significance. Second is the inherent variability of digital templating and surgical execution, which may potentially explain differences in the results. To decrease variability and detect templates that were not performed properly, we had 3 orthopaedic surgeons who were not part of the original template process and independently assess the templates. Each one was formally trained in THA templating using a video tool that has already been proven to enhance template quality [23]. In addition, only radiographs with a marker, and cases for which the acetabular component was within one size (negative control), were included. Furthermore, templating was performed on a low AP pelvis for all patients to standardize across institutions. Studies show that the AP pelvis undersize the mean femoral offset compared to the AP hip which may introduce error into templating as well [37]. However, because we used the same standardized image for templating STS and CTTS, our comparative results still hold true. In addition, given the added AP fit of the CTTS, future studies should consider the templating using the lateral view. Regarding surgical execution, both surgeons have an academic practice, performing DAA using the same technique, with more than 10 years of experience. We believe that even when eliminating variability seems difficult to achieve entirely in these types of studies, our approach decreased this limitation. Among the strengths of our study, to our knowledge, it is the first study evaluating accuracy for CTTS uniplanar digital templating.

## Conclusions

The accuracy of uniplanar digital templating for CTTS is significantly lower as compared to a predecessor STS design, with the template most often suggesting a size bigger than what fits in the operating room. These inaccuracies associated with templating CTTS using conventional uniplanar digital radiographs could lead to intraoperative fracture if the surgeon attempts to match the broach/stem to the template. Bi-planar templating using both AP and lateral radiographs or 3D preoperative templating with computed tomography should be considered, especially during the initial learning curve with these stems. Moreover, a surgeon should not be unduly concerned if their ultimate stem size is under that of the template if they have achieved axial and rotational stability.

## Conflicts of interest

Claudio Diaz-Ledezma is in the editorial board of the Journal of Arthroplasty. Erik N. Hansen is an unpaid consultant for Corin Ltd. William J. Hozack received royalties from Stryker; is a paid consultant for Stryker; and is in the medical/orthopaedic publications editorial/governing board of JOA. All other authors declare no potential conflicts of interest.

For full disclosure statements refer to https://doi.org/10.1016/j.artd.2025.101658.

## CRediT authorship contribution statement

**Claudio Diaz-Ledezma:** Writing – review & editing, Writing – original draft, Methodology, Investigation, Formal analysis, Data curation, Conceptualization. **Angel X. Xiao:** Writing – review & editing, Methodology, Investigation, Data curation. **Juan David Lizcano:** Writing – review & editing. **Erik N. Hansen:** Writing – review & editing, Conceptualization. **Camilo Restrepo:** Writing – review & editing. **William J. Hozack:** Writing – review & editing, Conceptualization.

## Appendix A Supplementary data

Conflict of Interest Statement for Diaz-Ledezma

Conflict of Interest Statement for Hansen

Conflict of Interest Statement for Hozack

Conflict of Interest Statement for Lizcano

Conflict of Interest Statement for Restrepo

Conflict of Interest Statement for Xiao
