# Supplementary material for: Two-Dimensional Preoperative Digital Templating is Less Accurate When Using a Collared Triple Taper Stem Versus a Single Taper Design
Source: Arthroplast Today. 2025 Mar 11;32:101658. doi: 10.1016/j.artd.2025.101658 (PMC11932654; doi:10.1016/j.artd.2025.101658)
Supplement: Conflict of Interest Statement for Restrepo [file mmc5.pdf]

Docusign Envelope ID: 5154F70F-923F-4FF2-8151-2A5A766B8186

CONFLICT OF INTEREST STATEMENT

American Association of Hip and Knee Surgeons

(Adopted from the American Academy of Orthopaedic Surgeons disclosure statement)

The following form **must be filled out completely and submitted by each author (example, 6 authors, 6 forms). All items require a response. If there is no relevant disclosure for a given item, enter "*None*."**

Manuscript Title. Two-dimensional preoperative digital templating is less accurate when using a collared triple taper stem versus a single taper design

1. Royalties from a company or supplier (The following conflicts were disclosed)
None

2. Speakers bureau/paid presentations for a company or supplier (The following conflicts were disclosed)
None

3A. Paid employee for a company or supplier (The following conflicts were disclosed)
None

3B. Paid consultant for a company or supplier (The following conflicts were disclosed)
None

3C. Unpaid consultants for a company or supplier (The following conflicts were disclosed)
None

4. Stock or stock options in a company or supplier (The following conflicts were disclosed)
None

5. Research support from a company or supplier as a Principal Investigator (The following conflicts were disclosed)
None

6. Other financial or material support from a company or supplier (The following conflicts were disclosed)
None

7. Royalties, financial or material support from publishers (The following conflicts were disclosed)
None

8. Medical/Orthopaedic publications editorial/governing board (The following conflicts were disclosed)
None

9. Board member/committee appointments for a society (The following conflicts were disclosed)
None

Each author must sign AND print or type his/her name, date and submit a separate form

In addition, one BLINDED Conflict of Interest form (no author names used) should be submitted per manuscript with all author disclosures.

DocuSigned by:
Camilo Restrepo
DDB62EF97B214C4...

Camilo Restrepo		09/24/2024
Author Name (Print or Type)	Author Signature	Date
