# Supplementary material for: Two-Dimensional Preoperative Digital Templating is Less Accurate When Using a Collared Triple Taper Stem Versus a Single Taper Design
Source: Arthroplast Today. 2025 Mar 11;32:101658. doi: 10.1016/j.artd.2025.101658 (PMC11932654; doi:10.1016/j.artd.2025.101658)
Supplement: Conflict of Interest Statement for Xiao [file mmc6.pdf]

CONFLICT OF INTEREST STATEMENT

American Association of Hip and Knee Surgeons

(Adopted from the American Academy of Orthopaedic Surgeons disclosure statement)

The following form **must be filled out completely and submitted by each author (example, 6 authors, 6 forms). All items require a response. If there is no relevant disclosure for a given item, enter "*None*."**

Manuscript Title

1. Royalties from a company or supplier (The following conflicts were disclosed) none

2. Speakers bureau/paid presentations for a company or supplier (The following conflicts were disclosed) none

3A. Paid employee for a company or supplier (The following conflicts were disclosed) none

3B. Paid consultant for a company or supplier (The following conflicts were disclosed) none

3C. Unpaid consultants for a company or supplier (The following conflicts were disclosed) none

4. Stock or stock options in a company or supplier (The following conflicts were disclosed) none

5. Research support from a company or supplier as a Principal Investigator (The following conflicts were disclosed) none

6. Other financial or material support from a company or supplier (The following conflicts were disclosed) none

7. Royalties, financial or material support from publishers (The following conflicts were disclosed) none

8. Medical/Orthopaedic publications editorial/governing board (The following conflicts were disclosed) none

9. Board member/committee appointments for a society (The following conflicts were disclosed) none

Each author must sign AND print or type his/her name, date and submit a separate form

In addition, one BLINDED Conflict of Interest form (no author names used) should be submitted per manuscript with all author disclosures.

Angel Xiao	Angel Xiao (signature)	9/26/2024
Author Name (Print or Type)	Author Signature	Date
